# Supplementary material for: Psychological distress symptoms among healthcare professionals are significantly influenced by psychosocial work context, Ethiopia: A cross-sectional analysis
Source: PLoS One. 2020 Sep 18;15(9):e0239346. doi: 10.1371/journal.pone.0239346 (PMC7500597; doi:10.1371/journal.pone.0239346)
Supplement: Attachment — Submitted filename: Responses of authors to reviewers.docx [file pone.0239346.s002.docx]

**Responses of authors to reviewers (References correspond to the clean version manuscript)**

| S.n | Referees | Question/comment | Responses | | Reference |
| --- | --- | --- | --- | --- | --- |
| o |  |  |  |  |  |
|  |  |  |  | |  |
| 1 | Reviewer | The manuscript is generally well written with clear descriptions | Thank you for the comments. | | Clean |
|  |  |  |  |  |  |
|  | #1 | in the study purpose. However, some minor points need to be | ❖ | We have now adhered to the defined | version |
|  |  |  |  |  |  |
|  |  | addressed in the revision. |  | abbreviations where relevant as per the | manuscript |
|  |  |  |  |  |  |
|  |  | 1. Please adhere to the use of defined abbreviations. |  | comments. |  |
|  |  |  |  |  |  |
|  |  | For example, in line 77, there is a "psychological distress" term. |  |  |  |
|  |  | However, the psychological distress has been defined as PD at |  |  |  |
|  |  | the very beginning of the manuscript. Line 73 also has the |  |  |  |
|  |  | similar problem. I do not point out all of them, the authors |  |  |  |
|  |  | should check by themselves throughout the manuscript. |  |  |  |
|  |  |  |  | |  |
|  |  | 2. Line 118. Here comes a term of "K10". After re-read the | Thank you for the suggestion. | | Page 6, line |
|  |  | manuscript, I can identify that the K10 indicates the Kessler | ⮚ | We have now defined it and corrected as per | 112 |
|  |  |  |  |  |  |
|  |  | Psychological Distress Scale. However, the authors did not |  | the suggestion. |  |
|  |  |  |  |  |  |
|  |  | define the K10 when they mention the Kessler Psychological |  |  |  |
|  |  | Distress Scale. |  |  |  |
|  |  |  |  | |  |
|  |  | 3. Line 119. Research cannot be counted. Therefore, the authors | Thank you for the comments. | | Page 6, line |
|  |  | should correct "researches" into "research". In the text, the | -We have corrected the word as per the | | 119 |
|  |  | associated factors are for instance sex, age, work experience, | suggestion | |  |
|  |  | education. |  |  |  |
|  |  |  |  |  |  |

|  |  | 4. Line 138. The Data quality control section is the biggest | ^❖^ Thank you for the comments. | | See | method | |
| --- | --- | --- | --- | --- | --- | --- | --- |
|  |  | problem in the manuscript. It is unclear why the authors define | ⮚ | We have now corrected and explained it as | section | |  |
|  |  |  |  |  |  |  |  |
|  |  | these actions as data quality control. The section reads like the |  | a language validity in data collection tools |  |  |  |
|  |  |  |  |  | ‘Data | |  |
|  |  | descriptions of language validity. Moreover, some descriptions |  | and variable measurements. |  |  |  |
|  |  |  |  |  |  |  |  |
|  |  |  |  |  |  |  |  |
|  |  | are confusing in the section. Specifically, back-translation | ^⮚^ And also we have now to removed | | collection | | |
|  |  | indicates translating local language back to English. I cannot |  | confusing sentences. | tools | | and |
|  |  | understand why the authors can back translate the English to |  |  |  |  |  |
|  |  |  |  |  |  |  |  |
|  |  | local language. Also, did the authors conduct a forward |  |  | variable | |  |
|  |  |  |  |  |  |  |  |
|  |  | translation? Moreover, ensuring language validity (i.e., make |  |  | measuremen | | |
|  |  | sure that the local language questionnaire is equivalent to the |  |  | t’ | page | 6 |
|  |  |  |  |  |  |  |  |
|  |  | English questionnaire) is not about data quality. The language |  |  |  |  |  |
|  |  | validity ensures the questionnaire validity. |  |  | from | | line |
|  |  |  |  |  |  |  |  |
|  |  |  |  |  | 124-126 | |  |
|  |  |  |  | |  | | |
|  |  | 5. Following the previous comment. If the authors want to | Thank you for the suggestion. | | See page 7, | | |
|  |  | present how they control the data, they should inform the | -We have now corrected as per the suggestion | | line 140-150 | | |
|  |  | readers how they make sure that the data input and data | provided. | |  |  |  |
|  |  | collections have no errors. |  |  |  |  |  |
|  |  |  |  | |  | | |
|  |  | 6. Table 1. Not everyone is familiar with the Ethiopian Birr. | Thank you for the comments. | | See page 10, | | |
|  |  |  |  |  |  |  |  |
|  |  | Please provide an exchange rate of the Ethiopian Birr with | ⮚ | We have now provided the exchange rate of | line 182 | |  |
|  |  |  |  |  |  |  |  |
|  |  |  |  |  |  |  |  |

| USD. |  | the Ethiopian Birr with USD at footnote. |  |
| --- | --- | --- | --- |
|  |  |  |  |
| 7. Also Table 1. I think that the footnote *a* should go earlier | ❖ | Thank you for the comments. We have | See page 10, |
|  |  |  |  |
| than footnote *b*. |  | now corrected as per the suggestion. | line 181 |
|  |  |  |  |

| 8. Table 3. I cannot understand why the authors mentioned |  | Thank you for the comments. | See page 12, |
| --- | --- | --- | --- |
|  |  |  |  |
| "Hosmer and Lemeshow test = 0.754 showed that the model | ❖ | One of the most frequent questions we get | line 211 |
| fitted well" here. Also, there is a superscript b for Others in the |  |  |  |
|  |  | about logistic regression is “How can we |  |
| Profession category. However, there is no footnote to explain |  |  |  |
|  |  | tell if our model fits the data?” Often the |  |
| the meaning of b. |  |  |  |
|  |  | questioner is expressing a genuine interest |  |
|  |  |  |  |
|  |  | in knowing whether a model is a good |  |
|  |  | model or a not-so-good model. Anyways |  |
|  |  | we have now removed from the footnote. |  |
|  | ^❖^ And also we have corrected the footnote | |  |
|  |  | that explain the meaning of b. |  |
